# Preoperative consecutive treatment with isoprenaline and adenosine is safe and reduces ischaemia-reperfusion injury in a porcine model of cardiac surgery with recent acute myocardial infarction

**DOI:** 10.1093/ejcts/ezaf120

**Published:** 2025-04-04

**Authors:** Sarah Smith, Igor Khaliulin, Ettorino Di Tommaso, Vito D Bruno, Thomas W Johnson, Eva Sammut, Daniel Baz-Lopez, Julia Deutsch, M-Saadeh Suleiman, Raimondo Ascione

**Affiliations:** Medical School, Faculty of Health and Life Science, University of Bristol, Bristol, UK; Medical School, Faculty of Health and Life Science, University of Bristol, Bristol, UK; Medical School, Faculty of Health and Life Science, University of Bristol, Bristol, UK; Medical School, Faculty of Health and Life Science, University of Bristol, Bristol, UK; Medical School, Faculty of Health and Life Science, University of Bristol, Bristol, UK; Medical School, Faculty of Health and Life Science, University of Bristol, Bristol, UK; Medical School, Faculty of Health and Life Science, University of Bristol, Bristol, UK; Veterinary School and Translational Biomedical Research Centre, Faculty of Health Science, University of Bristol, Bristol, UK; Medical School, Faculty of Health and Life Science, University of Bristol, Bristol, UK; Medical School, Faculty of Health and Life Science, University of Bristol, Bristol, UK

**Keywords:** Myocardial infarction, Cardio-protection, Isoprenaline, Adenosine, Cardiac surgery

## Abstract

**OBJECTIVES:**

The goal of this study was to assess the feasibility, safety and efficacy of consecutive treatment with isoprenaline/adenosine (ISO/ADE) in a pig model of myocardial infarction and cardiac surgery.

**METHODS:**

The final ISO/ADE dose was selected from a pilot study (*n* = 8). In the subsequent randomized trial, 16 pigs underwent cardiac magnetic resonance imaging 4 weeks after a myocardial infarction, then were randomized to either the ISO/ADE (*n* = 8) or the control (*n* = 8) group before undergoing cardiac surgery with 1 h recovery. Feasibility and safety end points included the method of ISO/ADE delivery, serial blood pressure, heart rate, pH, ${\text{HCO}}_{3}^{-}$, circulating lactate levels, troponin levels and arrhythmias. Biomarkers of efficacy included serial lactate levels and serial pO_2_ mean arterial-to-venous functional ratio along with histologic levels of glycogen, protein carbonyls, O_2_, CO_2_, ${\text{HCO}}_{3}^{-}$ and fibrosis. Postoperative rates of low cardiac output and death were also recorded.

**RESULTS:**

Cardiac magnetic resonance measures of myocardial infarction did not differ between the groups. The selected method of ISO/ADE delivery was feasible. At no time were all safety outcomes measured in the ISO/ADE group worse than those in the control group. ISO/ADE reduced circulating lactate levels, preserved the serial pO_2_ mean arterial-to-venous functional ratio and reduced tissue-based glycogen and protein carbonylation. No other differences were observed. Low cardiac output and death occurred in 3/8 (37.5%) and 2/8 (25%) control animals versus 0% in the ISO/ADE group.

**CONCLUSIONS:**

The therapy was feasible and safe and improved biomarkers of efficacy. ISO/ADE was not associated with any postoperative low cardiac output and deaths versus 37.5% and 25%, respectively, in the control group. A pilot human study is warranted.

## GRAPHICAL ABSTRACT

**Figure:**
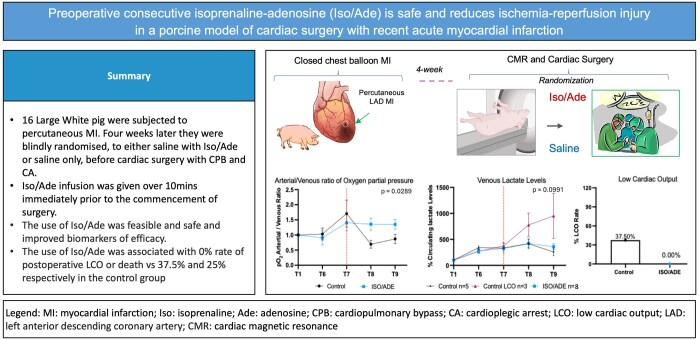

## INTRODUCTION

Globally, more than 64 million patients develop ischaemic heart failure following myocardial infarction (MI) [1]. Many of these patients are referred for coronary artery bypass grafting (CABG), which involves cardiopulmonary bypass (CPB) and cardioplegic arrest (CA), which can trigger ischaemia/reperfusion (I/R) injury and postoperative complications including low cardiac output (LCO) and death [2–4]. Myocardial protection during the operation remains challenging in these high-risk patients.

Temperature preconditioning [5] is cardioprotective against I/R injury because it increases myocardial cAMP levels, which in turn activate protein kinase A (PKA) and protein kinase C (PKC) [6]. We have shown in rat heart studies that triggering sequential cAMP/PKA/PKC activation with consecutive administration of isoprenaline and adenosine (ISO/ADE) protects the heart against I/R injury during both normothermic [6] and hypothermic CA [7]. ISO is used to treat bradycardia/heart block, LCO, hypovolemic/septic shock and/or cardiac arrhythmias [8–10]. ADE is used diagnostically in myocardial scintigraphy and therapeutically in patients with ventricular tachycardia (VT) [11]. The ISO dose for cardiogenic shock is 7–70 ng/kg/min (https://www.drugs.com/dosage/isoproterenol.html). The ADE dose to treat VT is a bolus of ∼40–170 µg/kg (https://www.drugs.com/uk/adenosine-3mg-ml-solution-for-injection-spc-11688.html). The rationale for using consecutive doses of ISO/ADE is that ISO, as a β-adrenergic receptor (β-AR) agonist, increases myocardial cAMP levels, which activate PKA and Epac (guanine-nucleotide exchange protein directly activated by cAMP) [12]. PKA and Epac activate PKC, which is expanded by the subsequent delivery of ADE [13]. Hence, the effect of ISO/ADE results from their synergistic effect on PKA and PKC myocardial levels when the 2 drugs are delivered sequentially [6].

The goal of this study was to ascertain the feasibility and safety of ISO/ADE in a complex porcine model of MI and cardiac surgery, relevant to high-risk patients with an MI undergoing CABG. We also assessed biomarkers of I/R injury.

## MATERIALS AND METHODS

Animal procedures were in accordance with the UK Home Office regulations (Animal Act 1986) under Project Licenses (7008975 and PP4585512), granted by the Home Office after approval by the local Animal Welfare and Ethics Review Body. Reporting of outcome was in line with ARRIVE (Animals in Research: Reporting In Vivo Experiments) guidelines [14]. Animal welfare was ensured by senior veterinary anaesthetists and animal technicians. The procedures were undertaken at the Translational Biomedical Research Centre (Bristol, UK) in line with good clinical practice (standard operating procedures) standards.

### Pilot pharmacokinetics study

Extended methods are provided in Supplementary Material, File. The doses tested were based on data from previous experiments in rat hearts [6, 15]. The goal was to study the pharmacokinetics, feasibility and safety of consecutive ISO/ADE infusion in 8 healthy female Large White pigs (∼60 kg) receiving 4 incremental doses (*n* = 2/group; Supplementary Material, Table S1) under general anaesthesia. Safety outcomes included serial heart rate (HR), systolic/diastolic blood pressure (SBP/DBP), circulating levels of glucose and Ca^2+^ and cardiac magnetic resonance (CMR) imaging indices. The goal was to select the highest ISO/ADE dose showing the least impact on safety outcomes. Drugs were prepared in saline with the ISO/ADE concentration ratio being ∼1:2,400 for each dose based on the ratio used previously on rat hearts [6, 15]. ISO/ADE was delivered during CMR imaging via a jugular venous line. The animals were monitored for 1 h before culling. Blood samples were collected at T1 (baseline), T2 (end of ISO infusion), T3 (end of ADE infusion) and T4 (20 min within the recovery period).

### Main feasibility/safety trial

**Figure 1:**
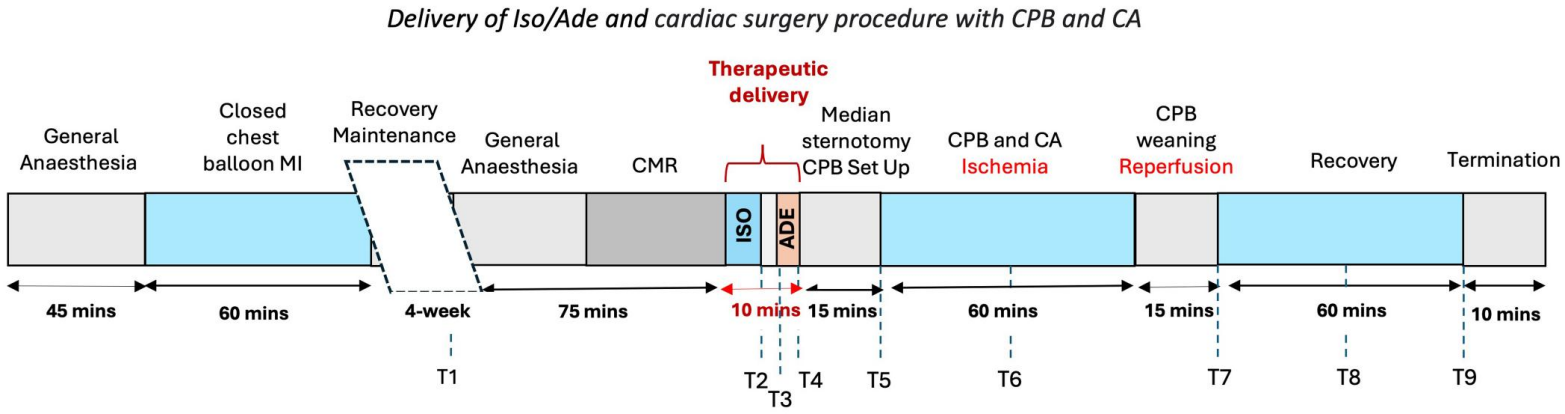
A schematic overview of the whole experiment. Predefined time points for serial vital parameter data included the following time points: T1 = baseline; T2 = end of isoprenaline infusion; T3 = end of flushing; T4 = end of adenosine infusion; T5 = start of cardiopulmonary bypass; T6 = 30 min of isoprenaline/adenosine ischaemia; T7 = end of cardiopulmonary bypass/reperfusion and start of recovery; T8 = 30 min into recovery; T9 = 60 min into recovery. Arterial and venous blood samples were collected concomitantly at T1, T6, T7, T8 and T9. ADE: adenosine; CA: cardiac arrest; CMR: cardiovascular magnetic resonance; CPB: cardiopulmonary bypass; ISO: isoprenaline; MI: myocardial infarction; T: time points.

A schematic of the whole experiment is shown in Fig. 1.

#### Porcine myocardial infarction procedure

Large White female swine weighing ∼60 kg (*n* = 18) were used. The MI procedure was as previously reported [16] (Supplementary Material, File). Briefly, with the pigs under general anaesthesia, heparinization (activated clotting time >300 s), statin and antiplatelet premedication, the left anterior descending coronary artery (LAD) was wired percutaneously via a 5F sheath inserted through the carotid artery. The LAD was occluded for 60 min distal to the first diagonal branch by inflating a 1:1–1.25 sized angioplasty balloon under fluoroscopic guidance. Prophylaxis and/or treatment of ventricular fibrillation was carried out with 300 mg amiodarone, administered intravenously starting at the coronary occlusion plus direct current cardioversion if necessary. After 60 min, the balloon was deflated and removed, and the animals recovered.

#### Randomization, isoprenaline/adenosine delivery, cardiac surgery and recovery

Four weeks after the MI, the surviving animals were randomized with 5/85 in each group undergoing CMR for MI characterization [17] before cardiac surgery (Supplementary Material, File). The animals were randomized according to 2 consecutive concealed block sequences, the first with 10 animals undergoing CMR and the second, with 6 animals with no CMR, to achieve *n* = 8 in each group. ISO/ADE or control infusions were prepared in concealed syringes before being taken to the operating room. The surgical and anaesthetic teams and those undertaking biomarker assays were kept blinded. The infusions were delivered over 10 min via a jugular venous line. The delivery method consisted of ISO or saline for 3 min, followed by 2 min of saline flushing, and then by a final infusion of ADE or saline for 5 min (Fig. 1).

Following a median sternotomy and heparinization (activated clotting time >400 s), CPB was established, and the temperature was allowed to drift to 35–36°C. Cardioplegic induction included 1 L of St Thomas antegrade cold blood cardioplegia in the aortic root with 2 maintenance doses (500 ml each) every 20 min to achieve 60 min of CA time. On completion, 15 min of heart reperfusion were allowed followed by CPB weaning, aiming for an SBP of 90–100 mmHg in all cases followed by 1 h recovery (Fig. 1). During recovery, a strict and blinded predefined resuscitation protocol was used. It included (i) re-infusing any residual blood from the CPB circuit; (ii) using small increments (0.5–1 mg) of metaraminol up to 3 mg; (iii) infusing up to 1 L of Normal Saline as additional volume replacement; and (iv) direct current cardioversion (up to 3 attempts) in case of VT.

#### Recording and terminating data points

Data points included T1 = baseline, T2 = end of isoprenaline infusion, T3 = end of flushing, T4 = end of adenosine infusion, T5 = start of CPB, T6 = 30 min of ischaemia, T7 = end of CPB and start of recovery, T8 = 30 min recovery and T9 = 60 min recovery (Fig. 1). Blood samples were collected concomitantly from arteries and veins at T1, T6, T7, T8 and T9. On completion of recovery, the animals were culled. Heart samples from infarcted and remote viable left ventricular regions of each heart were collected for comparative histologic analysis.

### Outcome measures

#### Pilot pharmacokinetics study

##### Safety outcome measures

Serial safety outcomes included HR, SBP and DBP, circulating levels of glucose, Ca^2+^ and all other variables determined from haematogas analysis (EPOC Portable Blood Gas and Critical Care Analyser, Woodley Equipment Company Ltd, Horwich, Bolton, UK). Left ventricular end-diastolic volume and stroke volume normalized for the pigs’ body weight (SVi) were measured via CMR imaging.

#### Main trial

##### Feasibility and safety outcomes measures

Feasibility focus was on delivering effectively the predefined method of consecutive ISO/ADE infusions. Safety measures included serial in vivo biomolecular markers of lactate, troponin levels, blood gas analysis, ST changes on electrocardiograms, asystole, heart block, marked hypertension and/or marked desaturation (peripheral oxygen saturation <90%). Concomitant arterial/venous levels of lactate, oxygen, carbon dioxide, bicarbonate and pH were measured at T1, T6, T7, T8 and T9 time points (Fig. 1). Troponin-I levels were measured at T1, T6, T7, T8 and T9 using a commercial ELISA kit (Life Diagnostics, Inc., CTNI-9-US. West Chester, PA, USA) according to the manufacturer’s protocol.

##### Biomarkers of efficacy

These biomarkers included serial circulating levels of lactate, serial pO_2_ mean arterial-to-venous functional ratio (pO_2_a-v f-ratio), histology-based glycogen, protein carbonyls, O_2_, CO_2_, ${\text{HCO}}_{3}^{-}$ and fibrosis levels. Postoperative occurrences of LCO and cardiac death were recorded. LCO was defined as severe haemodynamic deterioration or cardiac arrest during the postoperative 1 h recovery period despite strict implementation of the predefined resuscitation protocol. Histologic methods to test tissue-based biomarkers are reported in Supplementary Material, File.

### Statistical analysis and sample size

**Figure 2:**
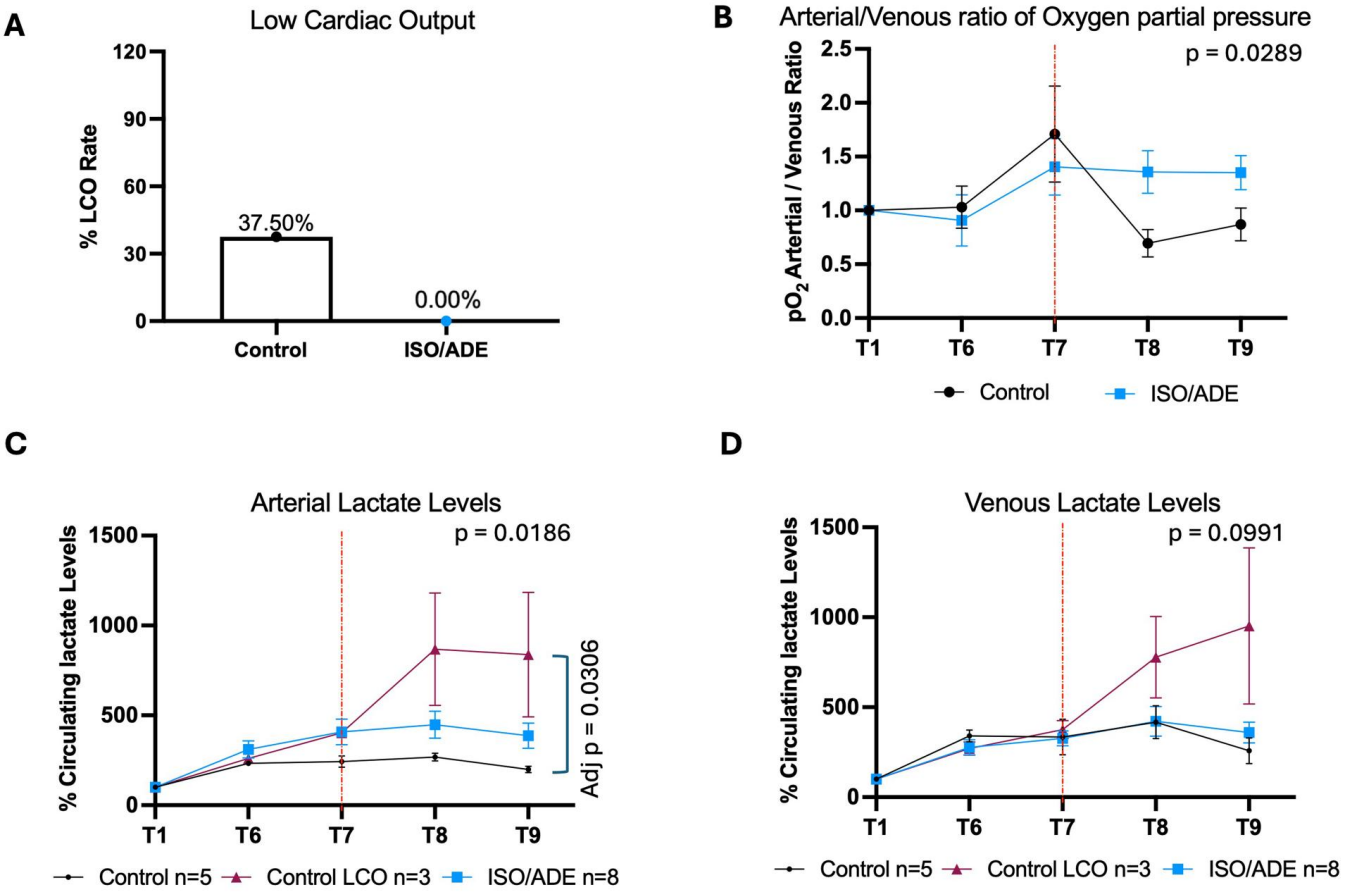
In vivo measures. (**A**) The low cardiac output rate for each experimental group presented as a percentage of the total number of animals per group (*n* = 8). (**B**) Partial pressure of oxygen, arterial: venous ratio expressed as a mean for each time point (*P* = 0.0289). (**C**) Arterial lactate levels expressed as a mean for each time point (*P* = 0.0186). Adjusted *P*-values of significance between groups (control vs low cardiac output, adjusted *P* = 0.0306; control vs isoprenaline/adenosine, *P* = 0.2218; low cardiac output vs ISO/ADE Adj*P* = 0.6156). (**D**) Venous lactate levels expressed as a mean for each time point (*P* = 0.0991). Adjusted *P*-values of significance between groups (control vs low cardiac output, adjusted *P* = 0.1065; control vs isoprenaline/adenosine, adjusted P > 0.9999; low cardiac output vs isoprenaline/adenosine, adjusted *P* = 0.2962). Area under the curve calculated for B–D from the start of recovery to the end of recovery (T7, T8, T9). Statistical analysis between groups was assessed using the Mann–Whitney test (B) or the Kruskal–Wallis test with multiple comparisons (C, D). *P* = level of significance. The red dashed line indicates the start of recovery following weaning from cardiopulmonary bypass. *n* = number per group = 8, unless otherwise stated in C, D. ADE: adenosine; AdjP: adjusted *P*-value; ISO: isoprenaline; LCO: low cardiac output.

Descriptive evaluation was undertaken for the pilot pharmacokinetics study as *n* = 2 in each subgroup. For the main trial, statistical analysis and graph design were performed using GraphPad Prism software 8.0.0 for Windows (San Diego, CA, USA; www.graphpad.com). Unpaired Student *t*-tests were performed with the alpha error set at 0.05. In Fig. 2B–D, the area under the curve (AUC) was assessed for each animal from the start of recovery to the end of recovery, which included 3 time points (T7, T8, T9) at which blood gas analyses was performed (refer to Fig. 1). To assess the AUC for each group in Fig. 2B, the Mann–Whitney test was applied to ensure an unbiased analysis of the variance between each group. In Fig. 2C and 2D, the Kruskal–Wallis test was applied due to the small subset of data (LCO) and to ensure a distribution free analysis of the data using Dunn’s multiple corrections tests. The *P*-values are shown on each graph, and adjusted *P*-values are detailed in the figure legends (Fig. 2C and D). Although most biomarkers were reported as measured values, in case of baseline variability across animals, the values of the subsequent time points were reported as percentages of the baseline value. Values in Table 1 and the figures are given as mean ± SEM where values greater than 0.05 significance are shown as *P* > 0.05. Because this was a feasibility and safety study, no formal sample size calculation was undertaken, although 8 animals were randomized in each group.

**Table 1:** Baseline characteristics

Variable	ISO/ADE (*n* = 8)	Control (*n* = 8)	*P*-value
Weight before MI (kg)	63 ± 1.75	63.48 ± 2.2	>0.05
Weight before cardiac operation (kg)	81.8 ± 2.39	82.75 ± 1.83	>0.05
Coronary occlusion (min)	60	60	NA
DC-treated VT/VF during MI	7/8 (87.5%)	8.8 (100%)	
CMR outcomes before cardiac operation	ISO/ADE (*n* = 5)	Control (*n* = 5)	
LV scar (g)	8.4 ± 2.39	8.8 ± 1.65	>0.05
LVEF (%)	46.2 ± 2.39	47.2 ± 2.31	>0.05
LVEDV (ml)	182 ± 12.7	186 ± 8.81	>0.05

CMR: cardiac magnetic resonance; DC: direct current; LV: left ventricle; LVEDV: left ventricular end-diastolic volume; LVEF: left ventricular ejection fraction; MI: myocardial infarction; VT/VF: ventricular tachycardia/ventricular fibrillation.

## RESULTS

### Pilot pharmacokinetics study

The HR rose in all 4 subgroups versus baseline during ISO infusion returning to baseline levels during ADE infusion (Supplementary Material, Fig. S1). The effect on SBP and DBP of all ISO/ADE doses was minimal over time with no differences across groups (Supplementary Material, Fig. S1). Levels of glucose and Ca^2+^ varied over time and versus baseline across the doses tested, with dose 4 showing the least variability (Supplementary Material, Fig. S1). CMR indices of left ventricular end-diastolic volume index (LVEDVi) and stroke volume index (SVi) did not differ across doses, although SVi appeared to improve with increasing doses, with dose 4 showing the largest effect (Supplementary Material, Fig. S1). On balance, dose 4 was selected for the main trial (ISO 83.33 ng/kg/min intravenously for 3 min followed by 2 min saline flushing and ADE 0.135 mg/kg/min intravenously for 5 min).

### Main experimental trial

MI procedures were performed in 18 animals; 2 of these who suffered untreatable ventricular fibrillation were excluded per the study protocol. The remaining 16 animals reached the post-MI 4-week time point and were randomized to ISO/ADE dose 4 (*n* = 8; ISO/ADE) or saline (*n* = 8; control), with *n* = 5 in each group undergoing CMR. Baseline weight, MI procedure aspects and CMR measures were similar among the groups (Table 1). After CMR and before opening the chest, all 16 animals were in very stable conditions with no need for any inotropic or vasopressors support. The average weight at 4 weeks was 82 kg, representing a gain of 18 kg versus baseline, with no difference among the groups (Table 1).

### Feasibility and safety outcome

ISO/ADE infusions were delivered according to plan in all cases. During the experiments, all predefined safety measures for HR, blood pressure, O_2_ saturation, lactate, troponin release and blood gas analysis in the ISO/ADE group were at no time worse than those observed in the control group (Supplementary Material, Table S2 and Fig. S3). Moreover, ISO/ADE did not trigger ST changes on the electrocardiogram, asystole, heart block, marked hypertension or low saturation.

### Biomarkers of efficacy

**Figure 3:**
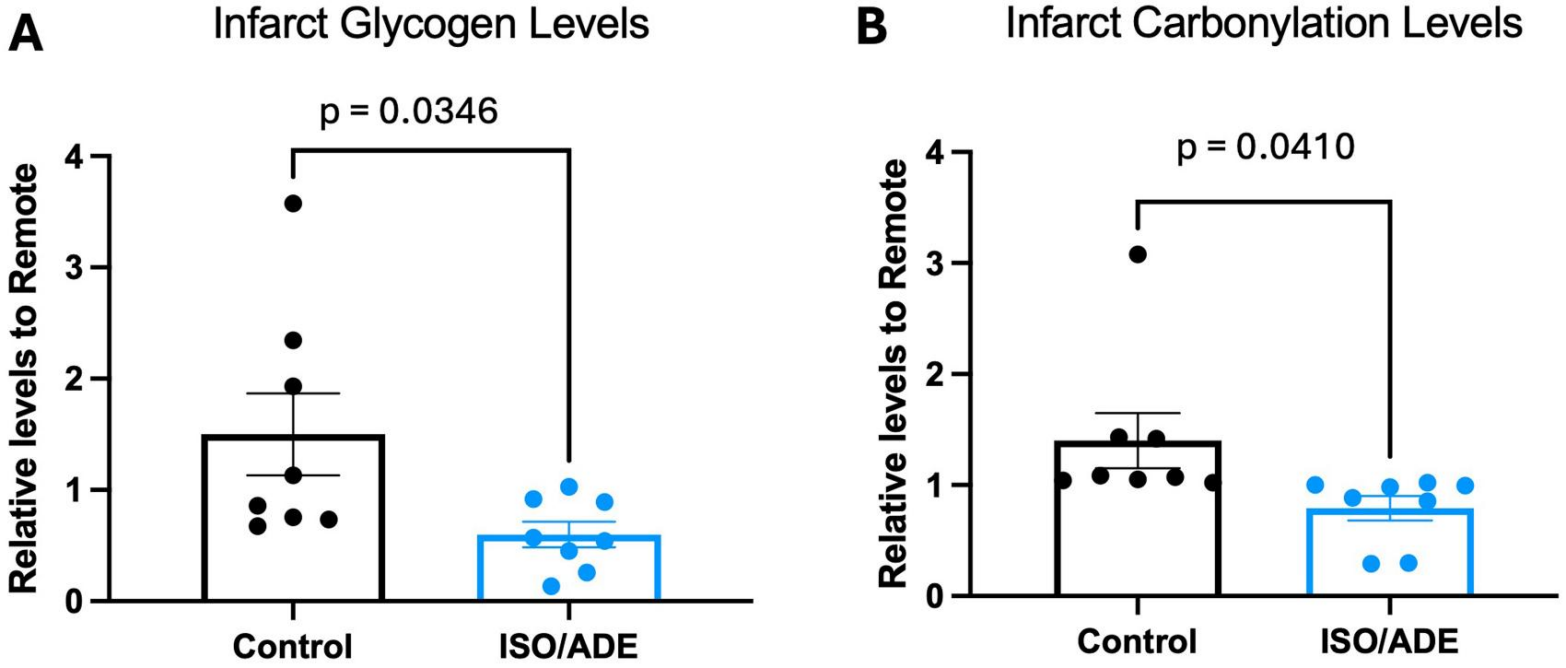
Tissue biomarker measures. (**A**) Total glycogen levels within infarct tissue relative to the remote region for each animal (*P* = 0.0346). (**B**) Protein carbonylation levels within infarct tissue relative to the remote region for each animal (*P* = 0.0410). *n* = 8 per group. ADE: adenosine; ISO: isoprenaline.

In vivo biomarkers are shown in Fig. 2. The index pO_2_a-v f-ratio was worse in the control group than in the ISO/ADE group (*P* < 0.029) (Fig. 2B). There was a 16.7% decrease in the AUC from cardiac reperfusion (T7) to the end of recovery (T9) and 13.6% decreases from T1 to T9 in the control group versus the ISO/ADE group, respectively. Levels of venous and arterial lactate were higher in the 3 LCO cases versus the 5 non-LCO cases in the control group versus the ISO/ADE group (both *P* < 0.05) (Fig. 2C and D). There was an 18% increase in the AUC of venous lactate from T7 to T9 and a 15.3% increase in the AUC from T1 to T9 for the control group versus the ISO/ADE group, respectively. There was a 4.5% increase in the AUC in arterial lactate from T7 to T9 in the control versus ISO/ADE groups, respectively. ISO/ADE was associated with reduced tissue levels of glycogen and carbonyls within the infarcted versus the viable remote region compared to the control group (both *P* < 0.05) (Fig. 3). The amount of fibrosis/scarring across the infarcted LAD territory was similar between the groups (Supplementary Material, Fig. S2).

### Postoperative low cardiac output and death

LCO was observed postoperatively in 3/8 (37.5%) animals in the control group (2/3 LCO cases had received CMR before surgery) versus 0/8 (0%) in the ISO/ADE group (Fig. 2A). In addition, 2/8 deaths (25%) occurred in the control group among the 3 LCO cases versus 0/8 deaths (0%) in the ISO/ADE group. The 2 deaths were due to persisting LCO and hypotension and occurred, despite strict and blinded adherence to the resuscitation protocol, at 20 and 40 min into the recovery period.

## DISCUSSION

This study confirms that the use of ISO/ADE is feasible and safe. In addition, ISO/ADE was associated with improved biomarkers of myocardial I/R injury. The rates of postoperative LCO and death were 37.5% and 25.0% in the control group versus 0% in the ISO/ADE group. A rigorous study design was used with MI confirmation via CMR, randomization, blinding, clinical grade cardiac surgery and adherence to ARRIVE guidance [14] for data reporting.

Regarding safety, a mild increase in HR occurred only during the 3 min of ISO infusion with prompt recovery to baseline levels. This result is in keeping with the results of previous studies on rat hearts [18] and those of a human study in which 30 min of ISO infusion at 5–15 ng/kg/min triggered transient dose-dependent increase in HR, SBP and insulin levels [19]. Moreover, 15 min intracoronary infusion of ISO at 12 ng/kg/min in pigs increased the left ventricle dP/dt and HR by 24 bpm [16]. Noticeably, none of the safety measures tested was worse in the ISO/ADE group than in the control group. These encouraging findings were obtained in a complex porcine model relevant to high-risk patients with an MI undergoing CABG and can help make the case for a future human pilot study. Little clinical research has been carried out in this area, with preoperative MI and impaired left ventricular function still being regarded as predictors of postoperative LCO and death following CABG [20, 21] and being reflected in the EuroSCOREs [22] and the Society of Thoracic Surgeons scores [23] and recommendations to avoid CABG in the early period post-MI [20, 21].

ISO/ADE reduced serial lactate levels and preserved pO_2_a-v f-ratio levels. High levels of blood lactate are a sign of poor organ perfusion associated with impaired cardiac function, a condition that can also lead to reduced levels of the pO_2_a-v f-ratio [24]. Indeed, a deranged pO_2_a-v f-ratio is typical of cardiogenic shock, reflecting impaired cardiac function and systemic organ hypoperfusion [22, 24, 25]. ISO/ADE was associated with reduced tissue levels of glycogen and protein carbonyl, both regarded as signs of biomolecular efficacy. Indeed, although a chronic drop in glycogen may be deleterious [26], given its role as a source of reserve energy, glycogen depletion prior to acute ischaemia is cardioprotective because it reduces the dissociation of hexokinase 2 from mitochondria and therefore inhibits the opening of mitochondrial permeability transition pores [6, 15, 27]. Hence, ISO/ADE being delivered ∼30 min before CA in the present study might have triggered a glycogen drop that protected the heart from the subsequent CA-related acute ischaemia. Concomitantly, the reduction in protein carbonyl is another biomolecular sign of efficacy, representing a reduced level of oxidative protein damage [28].

Cumulatively, the confirmation of feasibility and safety, the biomolecular signs of efficacy and the absence of postoperative LCO and death warrant a future pilot trial testing ISO/ADE in MI patients undergoing CABG. The dose used in this study is in keeping with dose ranges already used in clinical applications [10, 11] and could be easily modulated by patient weight, thereby making the use of ISO/ADE in humans feasible regardless of weight variations among patients.

The use of a combined model of MI and cardiac surgery might have helped to confirm the safety of ISO/ADE, given that small rodent models recapitulate poorly the myocardial I/R injury of MI patients undergoing cardiac surgery. The structures of porcine coronary arteries are similar to those of the human heart, making porcine MI models predictable in terms of infarct size, with cardiac kinetics and healing processes being similar [29, 30].

The sequential delivery of ISO/ADE is important because mixing these drugs within a single delivery is considerably less protective [6]. The duration and doses used are also critical for the effect of the β-AR on the myocardium. Whereas heart injury via sustained excessive β-AR stimulation can trigger arrhythmias [31], heart failure [32] and cell death [32, 33], brief and moderate β-AR stimulation has been associated with cardioprotection by our group [5, 6, 18, 34] and others [33, 35]. With the consecutive ISO/ADE treatment, the β-AR agonist ISO briefly elevates the myocardial cAMP levels, thereby activating PKA reported to be cardioprotective via mitochondrial permeability transition pore inhibition by GSK-3β phosphorylation [36]. PKA can also activate PKC via increased reactive oxygen species production [37] and the accumulation of intracellular Ca^2+^ [38]. Another downstream target of cAMP, Epac, can also activate PKC independently of PKA [39]. Infusing ADE after ISO triggers additional activation of PKC. Previously, we showed that inhibition of PKA by H-89 was able to prevent both PKC activation and cardioprotection [6] whereas the PKC inhibitor chelerythrine abrogated temperature preconditioning-induced cardioprotection [5].

It might be argued that 60 min of CA time was too short. However, the CA time in patients undergoing a typical CABG × 3 procedure is around 60 min. It might also be suggested that the 1 h recovery period was too short. However, we focused on the early period after CPB weaning, which is often when inotropic support, intra-aortic balloon pump and/or left ventricular assist devices are used. Another limitation of this study could be the small sample size. However, this was a safety trial, and the sample size was in keeping with data from a large systematic review on large-animal studies reporting most of the sample sizes from 6 to 10 animals in each group [40].

## CONCLUSION

In conclusion, the consecutive ISO/ADE therapy was feasible and safe. In addition, it improved biomarkers of I/R injury. The use of ISO/ADE was associated with 0% postoperative rates of LCO and death versus 37.5% and 25%, respectively, in the control group. These results warrant further testing in a pilot human trial.

## Supplementary Material

ezaf120_Supplementary_Data

## Data Availability

Full raw data and trial materials can be made available on reasonable request.

## ABBREVIATIONS

ADE: Adenosine
AUC: Area under the curve
β-AR: β-adrenergic receptor
CA: Cardioplegic arrest
CABG: Coronary artery bypass grafting
CMR: Cardiac magnetic resonance
CPB: Cardiopulmonary bypass
DBP: Diastolic blood pressure
Epac: Guanine-nucleotide exchange protein directly activated by cAMP
HR: Heart rate
I/R: Ischaemia/reperfusion
ISO: Isoprenaline
LAD: Left anterior descending coronary artery
LCO: Low cardiac output
MI: Myocardial infarction
PKA: Protein kinase A
PKC: Protein kinase C
pO_2_a-v f-ratio: Serial pO_2_ mean arterial-to-venous functional ratio
SBP: Systolic blood pressure
SpO_2_: Peripheral oxygen saturation
SVi: Stroke volume normalized for the pigs’ body weight
VT: Ventricular tachycardia
