# Supplementary material for: Preoperative consecutive treatment with isoprenaline and adenosine is safe and reduces ischaemia-reperfusion injury in a porcine model of cardiac surgery with recent acute myocardial infarction
Source: Eur J Cardiothorac Surg. 2025 Apr 4;67(5):ezaf120. doi: 10.1093/ejcts/ezaf120 (PMC12057998; doi:10.1093/ejcts/ezaf120)
Supplement: ezaf120_Supplementary_Data [file ezaf120_supplementary_data.docx]

## Supplementary file

**Extended Materials, Methods, and Results.**

*Pilot PK study to select Iso-Ade dose for the main trial*

The selected dosage of isoprenaline (Iso) and adenosine (Ade) was based on previous experiments on isolated Langendorff-perfused rat heart (6, 7). This work showed that the most effective dose was 10 nM of Iso infused via the aorta over 3 min (equivalent to ~70 ng/kg/min) followed by a 5 min infusion of Ade (30 µM; equivalent to ~270 µg/kg/min) ^1^. Four incremental Iso and Ade doses were prepared via dilution with saline solution and infused at the rate of 10 ml/min over the pre-defined time periods as detailed here:

**Dose 1**.

5 µg of Iso (~30 ng/kg/min) IV infusion for 3 min followed by 2 min of saline flushing and then by 12 mg of Ade (44 µg/kg/min) IV infusion over 5 min.

- - 0.1 ml of Isoprenaline Sulphate for injections (500 µg/ml) in 29.9 ml of saline
- 4 ml of Adenoscan^®^ for injections (3 mg/ml) in 46 ml of saline

**Dose 2**.

7.5 µg of Iso (45 ng/kg/min) IV infusion for 3 min followed by 2 min of saline flushing and then by 18 mg (88 µg/kg/min) IV infusion of Ade over 5 min.

- 0.15 ml of Isoprenaline Sulphate for injections in 29.85 ml of saline
- 6 ml of Adenoscan^®^ for injections (3 mg/ml) in 44 ml of saline

**Dose 3**.

10 µg of Iso (60 ng/kg/min) IV infusion over 3 min followed by 2 min of saline flushing and then by 27 mg of Ade (198 µg/kg/min) IV infusion over 5 min.

- 0.2 ml of Isoprenaline Sulphate for injections (500 µg/ml) in 29.8 ml of saline
- 8 ml of Adenoscan^®^ for injections (3 mg/ml) in 42 ml of saline

**Dose 4**.

15 µg of Iso (83.33 ng/kg/min) IV infusion over 3 min followed by 2 min of saline flushing and then by 40.5 mg of Ade (0.135 mg/kg/min) IV infusion over 5 min. Example for a 60kg pig:

- 0.3 ml of Isoprenaline Sulphate for injections (500 µg/ml) in 29.7 ml of saline
- 13.5 ml of Adenoscan^®^ for injections (3 mg/ml) in 36.5 ml of saline

We used a dose close to the most effective dosage derived from the isolated rat heart model scaled up for the targeted pig weight. Also, we planned to test 2 lower and 1 higher doses than the dose scaled up based on results from the rat heart experiments (Table S1).

**Table S1.**

| Dose | Isoprenaline (ng/kg) | Adenosine (mg/kg) |
| --- | --- | --- |
| 1 | 83 | 0.2 |
| 2 | 125 | 0.3 |
| 3 | 167 | 0.45 |
| 4 | 250 | 0.68 |

**Results of PK study**


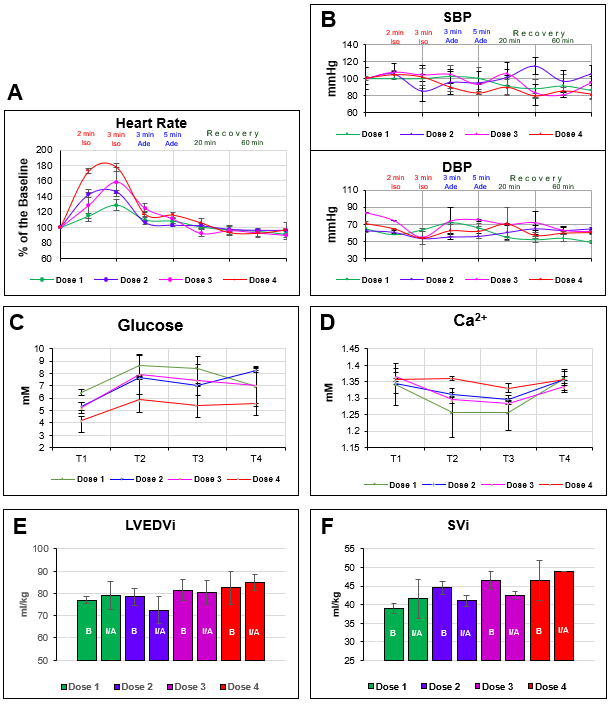


***Figure S1. Changes of vital parameters, levels of glucose and Ca^2+^ and cardiac function overtime****.*

*Changes of HR (****A****), and systolic (SBP) or diastolic (DBP) blood pressure (****B****) during 3 min Iso infusion, 5 min Ade infusion, and 60 min of recovery period across the four doses tested. Circulating levels of glucose (****C****) and Ca^2+^ (****D)*** *during the Iso/Ade treatments. Changes of left ventricular end-diastolic volume (LVEDVi)(****F)*** *and stroke volume (SVi) at CMR imaging normalized for the animals’ body weight, at baseline (B) and following the Iso/Ade treatment (I/A) for each Iso/Ade dose.*

***Randomization and blinding***

Animals were randomized following CMR and before cardiac surgery according to a concealed block randomisation sequence based on 2 consecutive blocks, one of 10 animals (with CMR) and the second one of 6 animals (no CMR) to achieve a balanced distribution of n=8 between groups. The infusions of Iso/Ade or control were prepared in concealed syringes in the adjacent lab and taken into the surgical theatre few minutes before they were due. The surgical and anaesthetic teams were kept blind along with those undertaking lab assays and histology testing.

***Porcine MI procedure***

Large Female White swine weighing ~62 kg (n=18) was used. The MI procedure was carried out as previously reported (19). Briefly, under general anaesthetic (GA), full monitoring, heparinisation (activated clotting time > 300 sec), and antiplatelet pre-medication (aspirin 300 mg daily with food starting 5 days before MI until termination), the left anterior descending coronary artery (LAD) was wired percutaneously via a 6F sheath inserted through the right carotid artery. The LAD was occluded for 60 min distal to the first diagonal branch to start ischemia using a 1:1-1.25 sized angioplasty balloon (Ryujin balloon, Terumo, Japan) under fluoroscopic guidance (Siemens Artis Zee, floor mounted). Amiodarone infusion (300 mg over 90 min) was started just before coronary occlusion to prevent ventricular fibrillation (VF). Additional bolus (150 mg) and direct current (DC) cardioversion were used to treat VF (if any) during ischemia. On completion of 60 min, the coronary balloon was deflated (reperfusion confirmed under angiographic check) and removed. Animals were recovered for 45-60 min under monitoring of key vital parameters and then moved to the maintenance area for 4 weeks.

*Delivery of Iso/Ade and cardiac surgery procedure with CPB and CA*

At 4-weeks following the MI procedure, all surviving animals were subjected to GA and continuous monitoring of electrocardiogram (ECG), BP, HR, temperature, central venous pressure, and urine output. Next, animals were randomised and 5 pigs in each group underwent CMR imaging for baseline MI characterization using a 3T scanner with a 32‐element phased‐array cardiac coil (Siemens PRISMA, Germany). CMR protocol included cine sequences in long and short axis, pre-contrast T2 mapping, pre-contrast (native) and post-contrast T1 mapping and extracellular volume (ECV) measurements, and early and late gadolinium enhancement (19). Image acquisition was performed by a blinded expert CMR radiographer while CMR analysis was performed by 1 independent blinded experienced investigator using dedicated software (CVI42, Circle, v5.13.9). Iso/Ade or saline (Control) IV infusions were delivered blindly via a venous line inserted in the jugular vein before starting CPB. Infusions were over 10min in all cases and consisted of either Iso or saline over 3 min, followed by 2 min of saline flushing, and then by a final IV infusion of either Ade or saline over 5 min. Following median sternotomy and heparinisation (starting dose 20,000 units, to achieve an activated clotting time (ACT) > 400 sec), CPB was established with a double-stage cannula from the right atrium for drainage and a perfusion cannula in the distal ascending aorta. Once on CPB, the body temperature was allowed to drift to 35-36°C, the aortic cross-clamp was applied and the heart was stopped in all cases with 1 L of St Thomas antegrade cold blood cardioplegia delivered via the aortic root plus topical ice. Two additional doses of cardioplegia (500 mL each) were delivered every 20 min (plus topical ice) to achieve a total CA time of 60 min. On completion, the aortic cross-clamp was removed to allow a 15min reperfusion along with restarting of mechanical ventilation. On completion of reperfusion, the CPB was weaned aiming for a systolic blood pressure within the 90-100 mmHg range. The heart was then decannulated to start a final period of 1h recovery. During the recovery period, a strict predefined volume replacement/resuscitation protocol was used blindly aiming at keeping the targeted SBP using the following resuscitations methods: **1**. Re-infusing any residual blood from the CPB circuit, **2**. using small increments (0.5-1 mg) of metaraminol up to a maximum of 3 mg, **3**. infusing a maximum of 1 L of N/Saline as additional volume replacement, and **4**. DC cardioversion (up to 3 attempts) in case of ventricular tachycardia (VT)/VF. Predefined time points for recording of serial vital parameter included: T1 = Baseline, T2 = End of isoprenaline infusion, T3 = End of flushing, T4 = End of adenosine infusion, T5 = Start of CPB, T6 = 30 minutes of Ischemia, T7 = End of CPB and Start of Recovery, T8 = 30 minutes Recovery, T9 = 60 minutes Recovery. Blood samples were collected concomitantly from arteries and veins at time points T1, T6, T7, T8, and T9 . On completion of 1h recovery, the animals were culled and heart samples from infarcted and remote viable left ventricular (LV) regions of each heart were collected for histopathology and biochemical evaluations.

*Histological methods*

Myocardial samples were collected from the infarcted and remote viable territories for each heart soon after culling. For tissue glycogen and protein carbonylation assays, tissue was rapidly homogenised with a pestle and mortar in LN_2_. Total glycogen levels were determined and quantified using Glycogen Assay Kit II, Colorimetric (Abcam, ab169558), following manufacturer’s standard protocol. Tissue protein carbonyl levels were measured using a protein carbonyl assay kit (Sigma, MAK094) following the manufacturer’s standard protocol.  Tissue was also processed and paraffin-embedded. Three distinct areas of each infarcted and remote viable region were cut at 5 μm thickness, stained with haematoxylin and eosin (H&E). Picrosirius red stain was for fibrosis/collagen analysis. Stained tissue was imaged using a light microscope at x20 magnification. Staining per tissue section was determined using colour deconvolution in Fiji ImageJ (Java 1.8.0 64-bit) and expressed as an average percentage of the cardiac cross-section area for each animal.

**Results**


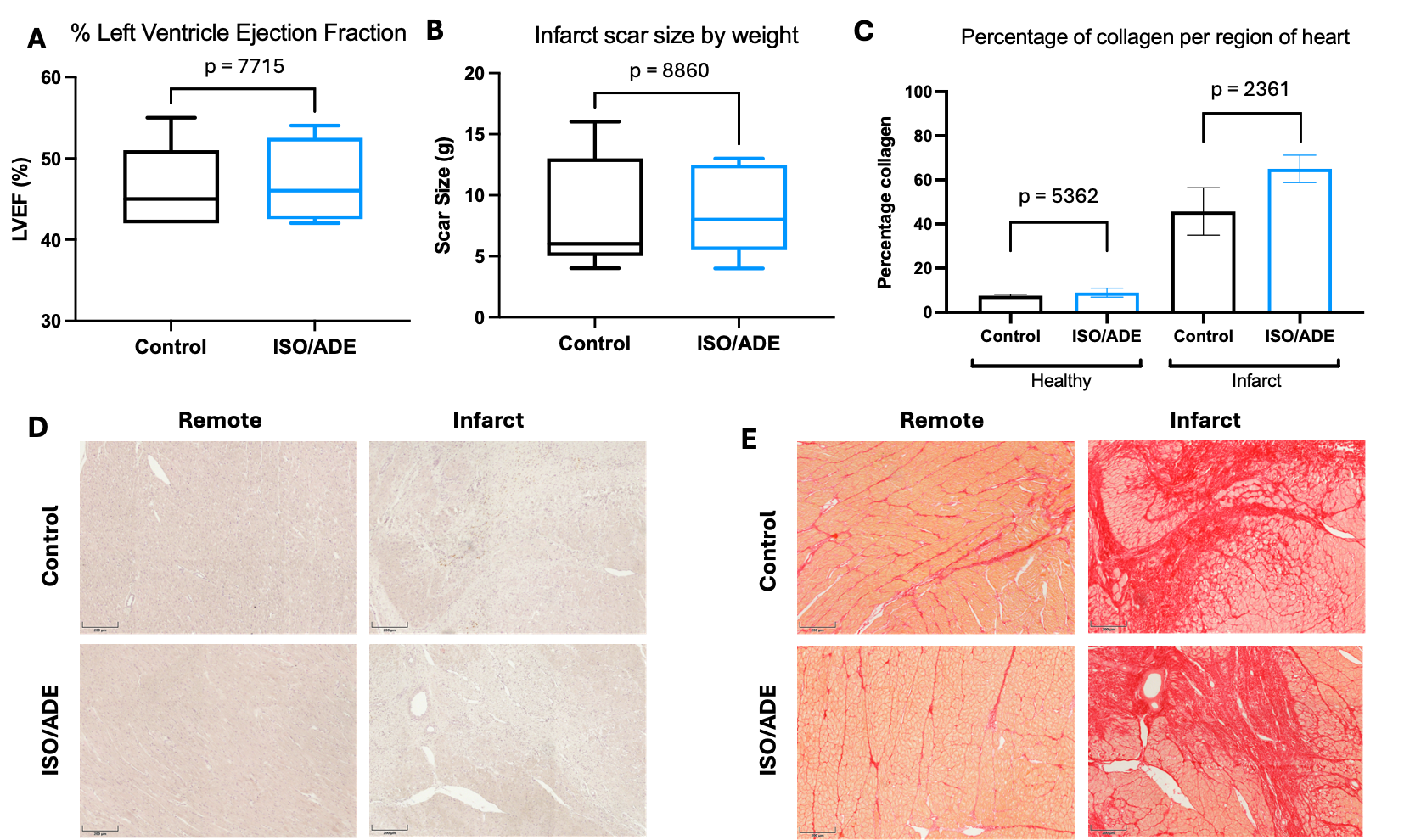


##### ***Figure S2. Comparison of LV function, LV scar, and basic histology between groups****. A) Mean percentage of left ventricular ejection fraction (LVEF) of both groups (n=5 each); B) Mean LV scar size in grams of both groups (n=5 each); C) Histology quantification of collagen levels in viable remote and infarcted regions. D) Representative images of H&E stain in viable remote and infarcted regions per group. x20 magnification, n=8. E) Representative images of picrosirius red staining for total collagen in remote and infarct regions for each group. x20 magnification (quantification in C), n=8. P-values for A-C) = > 0.05.*

*Troponin levels*

Levels of pig cardiac troponin did not rise in the Iso/Ade group during surgery or the 1 h recovery compared with the control (Figure S3).

**Figure S3**

**
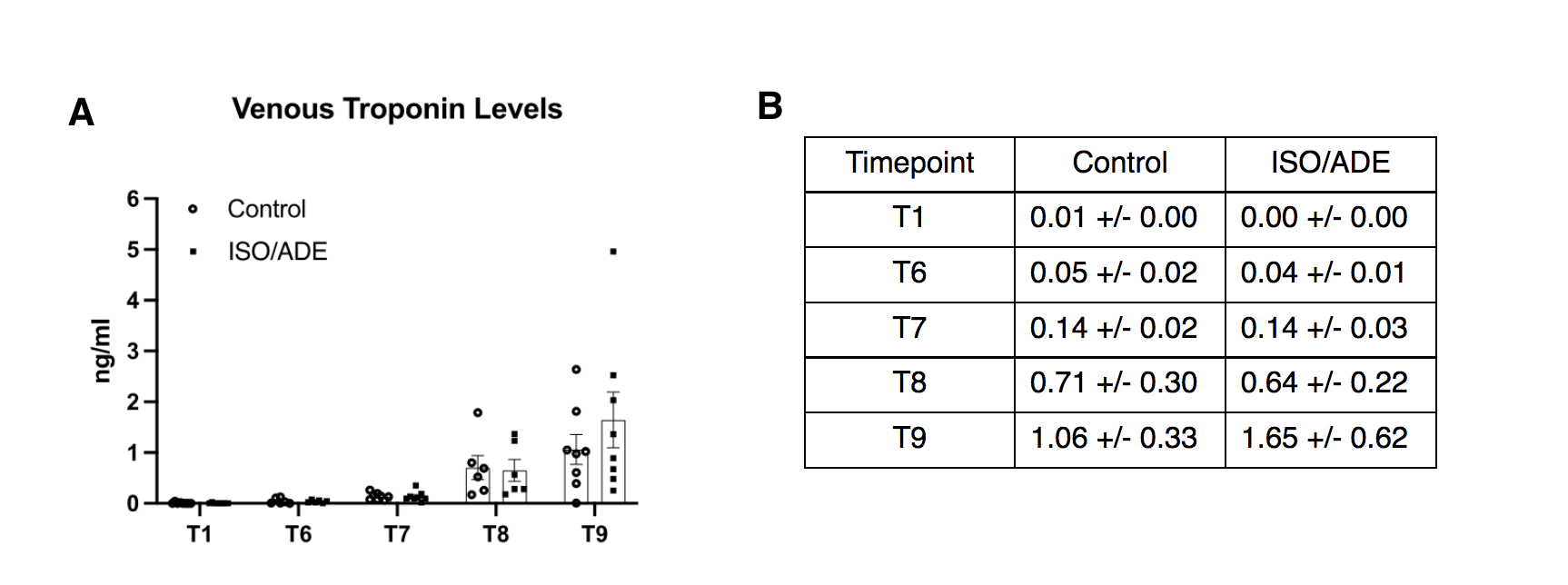
**

*A) Venous Blood Troponin Levels for ISO/ADE and Control group measured at timepoints T1, T6, T7, T8, and T9. B) Mean Troponin levels in ng/ml for each group at all time points with +/- = standard error of the mean.*

*Tissue outcome measures*

Following culling, the infarcted/scarred LAD territory was collected for each heart along with samples of the remote viable myocardium from the circumflex artery (CX) territory of each heart. Tissue was routinely processed, and paraffin-embedded. Three distinct areas of the remote viable region and infarcted/scarred region were cut at 5 μm thickness and sections stained with haematoxylin and eosin or Picrosirius Red (for collagen/fibrosis) for histopathological examination and imaged using a light microscope at x20 magnification.  Analysis was carried out using colour deconvolution in Fiji ImageJ (Java 1.8.0 64-bit).  In addition, tissue samples were lysed and prepared for glycogen and protein carbonyl examinations. Expanded methods can be found below.

*Tissue outcome measures*

*Picrosirius Red Staining*

Tissue was routinely processed, and paraffin-embedded. Three distinct areas of the remote region and infarct region for each animal were cut at 5 μm thickness and sections were stained with picrosirius red. Briefly, sections were placed in distilled H_2_O followed by 3 x 5 min exposure to Clearene, 2 x 5 minutes 100% IMS, 1 x 5 min 70% IMS, and 1 x 5 min distilled H_2_O. Sections were incubated in 0.1% picrosirius red solution at room temperature in the dark for 90 min. Sections were rinsed 2 x 5 min 0.01N HCL, 2 x 5 min distilled H_2_O, 2 x 1.5 min 100% IMS, and 3 x 5 min exposure to Clearene. The relative amount of collagen (red under a light microscope) was assessed using 5 images per section at x20 magnification, analysed using ImageJ software, and expressed as an average percentage of the cardiac cross-section area for each animal.

*Tissue Glycogen Assay*

Tissue samples were rapidly homogenised with a pestle and mortar in liquid nitrogen (LN_2_). Tissue was moved to 400 ml distilled H_2_0 on ice and then placed in a sonic homogeniser (3 x 10-sec pulse) prior to centrifugation at 12,000 x *g* for 15 min (4^O^C ) to remove insoluble material. The supernatant was transferred to a fresh Eppendorf tube and boiled (95°C) for 10 min to inactivate enzymes. Protein quantification was carried out using a standard BCA assay protocol (Thermo Fisher Scientific) and 50 mg of sample per well was used. Total glycogen levels were determined and quantified using Glycogen Assay Kit II, Colorimetric (Abcam, ab169558), following the manufacturer’s standard protocol.

*Tissue Protein Carbonyl Assay*

Tissue samples were rapidly homogenised with a pestle and mortar in LN_2_ and transferred to 400 ml ultrapure H_2_0 on ice and then placed in a sonic homogeniser (3 x 10-sec pulse) prior to centrifugation at 12,000 x g for 15 min (4°C ) to remove insoluble material. To remove nucleic acids, samples were incubated with 40 mL of the 10% streptozocin at room temperature for 15 min and then centrifuged 13,000 × g for 5 min with supernatant transferred to a fresh Eppendorf tube. Protein quantification was carried out using a standard BCA assay protocol (Thermo Fisher Scientific) and samples were diluted to 10 mg/mL. A protein Carbonyl Assay kit (Sigma, MAK094) was used to measure the levels of protein carbonylation according to the manufacturer’s standard protocol.


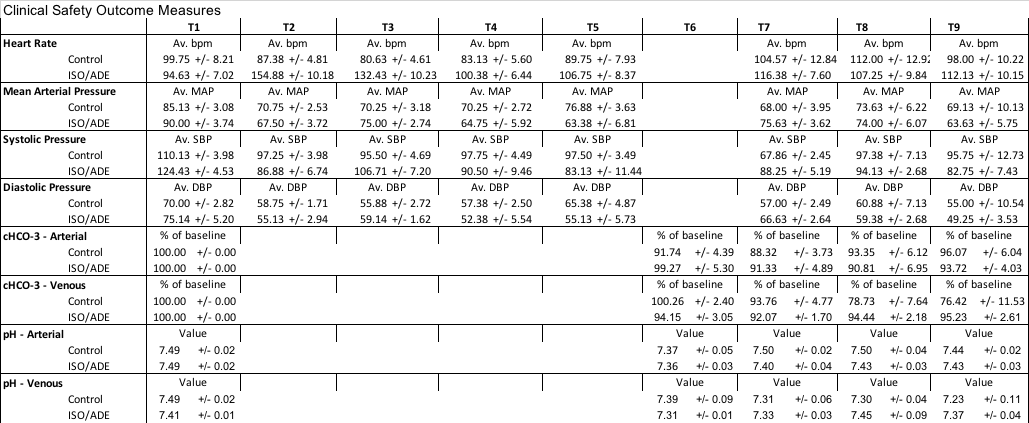
**Table S2.**
